# Sub–0.5-mm Resolution PET Versus Autoradiography: Comparison of mGluR1 Concentrations in Mouse Brain

**DOI:** 10.2967/jnumed.125.271600

**Published:** 2026-09

**Authors:** Han Gyu Kang, Hideaki Tashima, Hidekatsu Wakizaka, Takeharu Minamihisamatsu, Tomoteru Yamasaki, Masayuki Fujinaga, Ming-Rong Zhang, Makoto Higuchi, Taiga Yamaya

**Affiliations:** National Institutes for Quantum Science and Technology, Chiba, Japan

**Keywords:** preclinical PET, sub–0.5-mm resolution, autoradiography, ^18^F-FITM

## Abstract

For mouse brain PET imaging with high quantification accuracy, ultrahigh resolution is essential. Metabotropic glutamate receptors are important molecular targets, linked to various neurologic diseases. Here, we report the comparison of in vivo mouse brain images obtained with sub–0.5-mm resolution PET and those obtained with autoradiography using 4-^18^F-fluoro-*N*-[4-[6-(isopropylamino)pyrimidin-4-yl]-1,3-thiazol-2-yl]-*N*-methylbenzamide (^18^F-FITM) that binds to metabotropic glutamate receptor subtype 1. **Methods:** We recently developed a sub–0.5-mm resolution PET scanner having an axial coverage of 23.4 mm and an inner diameter of 48 mm. The PET scanner comprises 32 depth-of-interaction detectors, each of which has staggered 3-layer lutetium–yttrium–orthosilicate crystal arrays with a pitch of 0.8 mm. A resolution phantom was used to evaluate the imaging performance of the PET scanner. Dynamic PET imaging was performed to evaluate the concentration changes of ^18^F-FITM at various mouse brain regions. In vivo metabotropic glutamate receptor imaging of a mouse brain was performed using ^18^F-FITM and the PET scanner. Subsequently, autoradiography images of the same mouse brain were obtained using ^18^F-FITM. **Results:** The 0.45-mm rod structure was resolved with the PET scanner. Time–activity curves of various mouse brain regions were obtained. The sub–0.5-mm resolution PET visualized ^18^F-FITM uptake in the mouse brain with high correlation to the autoradiography images. **Conclusion:** The sub–0.5-mm resolution PET opens up new opportunities for translational neuroscience research using mice models.

## Visual Abstract

**Figure:**
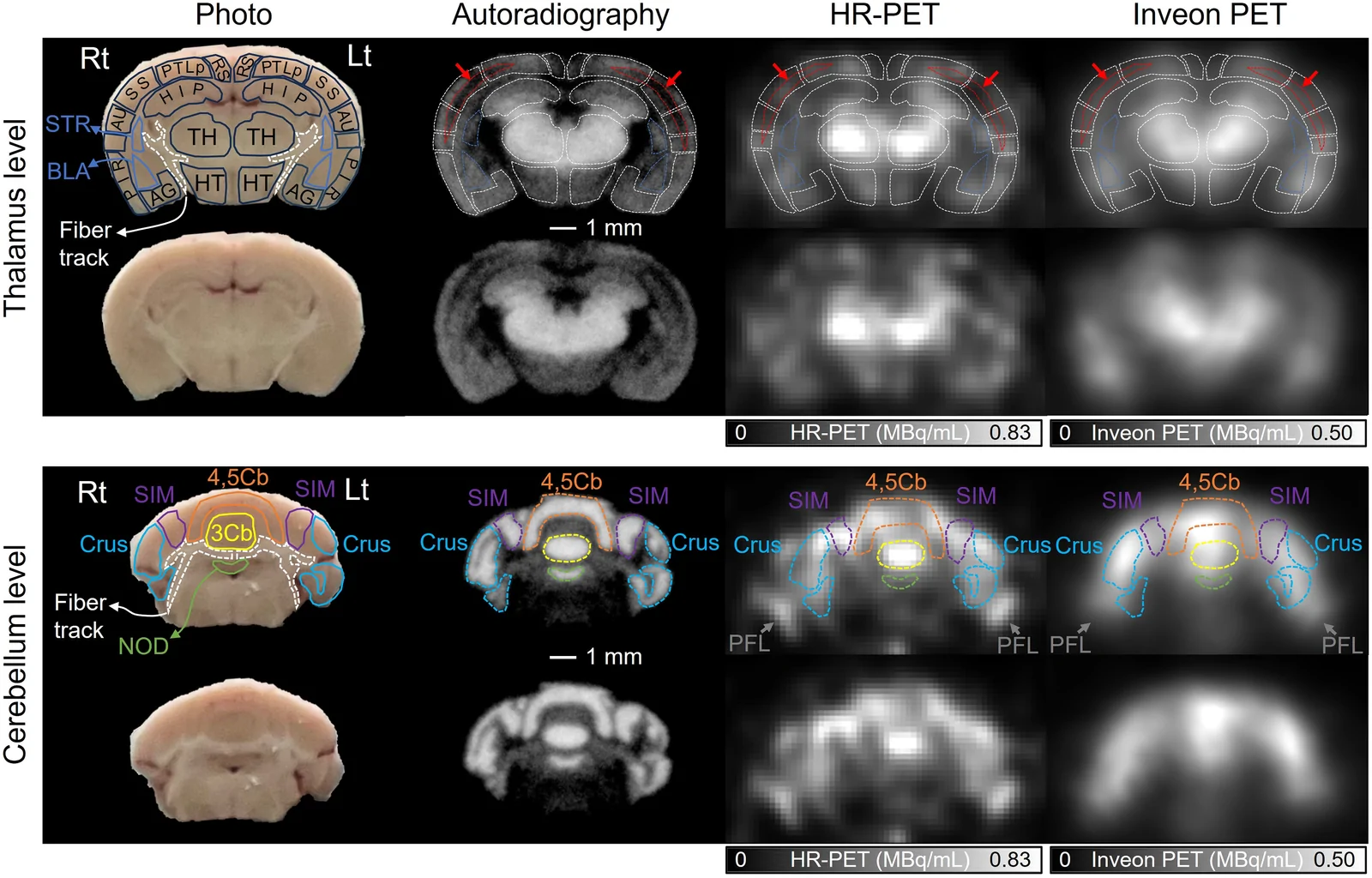

The PET ligand 4-^18^F-fluoro-*N*-[4-[6-(isopropylamino)pyrimidin-4-yl]-1,3-thiazol-2-yl]-*N*-methylbenzamide (^18^F-FITM) binds to metabotropic glutamate receptor subtype 1 (mGluR1), which is abundant in the cerebellar Purkinje cells, thalamus, and striatal interneurons of rodent models (1,2). The mGluR1s, G-protein–coupled receptors, are excited by glutamate, which is the primary excitatory neurotransmitter in the mammalian central nervous system. The mGluR1 signaling pathways affect motor control and learning, as well as long-term depression (3,4). Distribution and concentration of mGluR1s in the central nervous system are linked to various neurodegenerative diseases such as Alzheimer disease, Parkinson disease (5,6), and Huntington disease (7,8). Therefore, accurate imaging of mGluR1s in rodent brains is important for the development of new drugs to treat neurodegenerative diseases and disorders.

Autoradiography is the gold standard imaging technique to evaluate the distributions of PET tracers in rodent models with high resolution (a few tens of micrometers) (9). However, autoradiography does not allow multiple monitoring of radiotracer distributions at multiple time points for a single subject, as the subject should be sacrificed for the sectioning process. In addition, it is technically difficult and time-consuming to produce 3-dimensional (3D) images of the entire mouse brain with autoradiography.

On the other hand, PET allows in vivo 3D imaging of the entire mouse brain at multiple time points, thereby allowing monitoring of radiotracer uptake as a function of time. However, one critical issue in preclinical PET is the low spatial resolution of around 0.6 mm, which hampers visualization of small mouse brain regions (e.g., thalamus, hypothalamus, and cerebellar lobulus) (10–14). The spatial resolution of PET has been limited mainly because of large crystal pitch, crystal decoding accuracy, and depth-of-interaction (DOI) resolution. To address this issue, we recently developed a sub–0.5-mm resolution PET scanner with optimized staggered 3-layer DOI detectors (15,16). Physical performance was fully characterized (15); however, in vivo imaging performance with the ^18^F-FITM tracer was not compared against autoradiography. Therefore, here, we compare in vivo mouse brain images obtained by sub–0.5-mm resolution PET with those obtained by autoradiography when using the ^18^F-FITM tracer.

## MATERIALS AND METHODS

### Sub–0.5-mm Resolution PET Scanner

**FIGURE 1.**
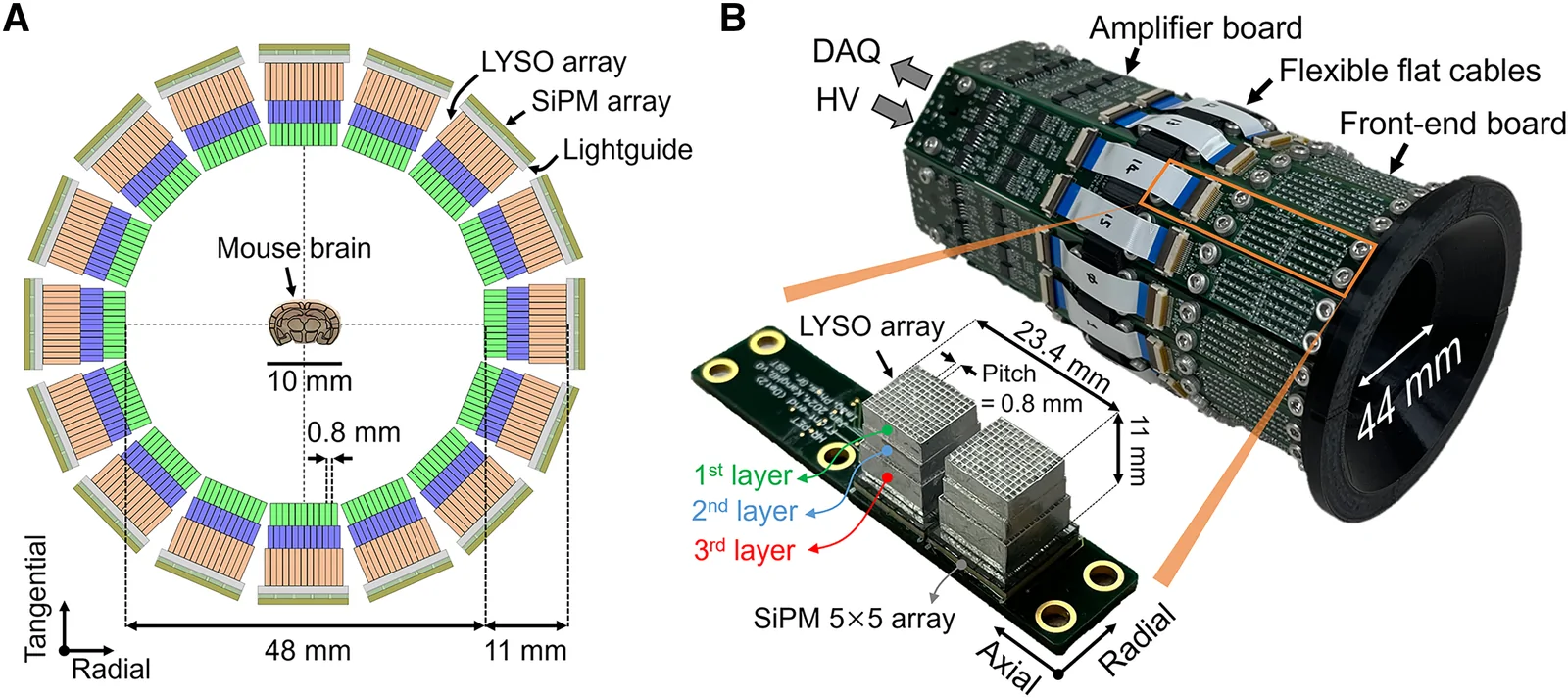
(A) Schematic front view of HR-PET. (B) Photo of HR-PET with one of the DOI detectors. DAQ = data acquisition; HV = high voltage; LYSO = lutetium–yttrium–oxyorthosilicate; SiPM = silicon photomultiplier.

The developed sub–0.5-mm resolution PET scanner (hereafter referred to as HR-PET) has a 23.4-mm axial field of view and a 48-mm inner-ring diameter (Fig. 1; Table 1) (15). The HR-PET has 2 rings, each of which has 16 DOI detectors. Each DOI detector has staggered 3-layer lutetium–yttrium–oxyorthosilicate crystal arrays, a 1-mm-thick acrylic light guide, and silicon photomultipliers in a 5 × 5 array with a 2.4-mm pitch (S13360-2050VE; Hamamatsu Photonics K.K.). Compared with our previous PET scanner (14), the crystal pitch was reduced from 1 mm to 0.8 mm. Individual crystals were optically isolated with a 0.1-mm-thick–powdered BaSO_4_ layer, which can provide better crystal decoding accuracy and DOI resolution than enhanced specular reflector films (17). The crystal thicknesses of the first, second, and third layers were 3, 3, and 5 mm, which were optimized with Geant4 Application for Tomographic Emission software (16). The multiplexed signals were digitized by a custom-made 160-channel data acquisition system with a sampling rate of 50 megasamples per second (15). For data correction, normalization and random correction were used. No scatter and attenuation corrections were used considering the small diameter of a mouse brain (10 mm). The details of physical and imaging performance were described previously (15).

**TABLE 1.** Specifications of the HR-PET (15)

Parameter	Value
Inner diameter (mm)	48
Axial coverage (mm)	23.4
Crystal pitch (mm)	0.8
First layer crystal size (mm^3^)	0.7 × 0.7 × 3.0 (11 × 12 array)
Second layer crystal size (mm^3^)	0.7 × 0.7 × 3.0 (12 × 12 array)
Third layer crystal size (mm^3^)	0.7 × 0.7 × 5.0 (13 × 13 array)
Silicon photomultiplier pitch (mm)	2.4 (5 × 5 array)
Energy resolution (%)	18.6
Coincidence timing resolution (ns)	8.5
Peak axial sensitivity (%)*	0.65
FBP spatial resolution (mm)^†^	0.67 ± 0.06
Resolvable rod diameter (mm)^‡^	0.45 (OSEM)

* Energy window was 440–560 keV.

† Radial resolution from center to radial offset of 10 mm using 2D FBP without gap filling.

‡ VPR was 0.679 ± 0.071 with a resolvability of 91.7% when using OSEM with 40 iterations and 8 iterations.

### Resolution Phantom Imaging

To evaluate imaging performance, we used a resolution phantom having 6 different rod diameters (0.45, 0.5, 0.55, 0.75, 0.8, and 0.85 mm) with a 10-mm-long rod length (Supplemental Fig. 1A; supplemental materials are available at http://jnm.snmjournals.org) (14,18). For each rod sector, the center-to-center distance between neighboring rods was twice the rod diameter. Each rod was filled with ^18^F solution, resulting in a total activity of 1.2 MBq. The phantom was scanned by the HR-PET for 30 min, which was half of our previous study (15) to make the scan time equal to that of the in vivo imaging study. Subsequently, coincidence events (6.6 million) were identified from single events by custom-written software with a coincidence window of 10 ns and an energy window of 440–560 keV, which were optimized in our previous study (15).

For analytic image reconstruction, we used 2-dimensional (2D)–filtered backprojection (FBP) with gap filling (16). For iterative image reconstruction, we used the list-mode 3D ordered-subset expectation maximization (OSEM) algorithm (15) with 8 subsets, 40 iterations, and 3D gaussian image domain blurring (kernel size, 0.55 mm) (Supplemental Table 1). The same voxel size (radial × tangential × axial) of 0.2 × 0.2 × 0.2 mm^3^ was used for FBP and OSEM algorithms. For data correction, we used normalization and random correction (15). The reconstructed phantom images were axially projected with a slice thickness of 6 mm (30 slices) to generate a summed PET image. For each rod sector, line profiles were extracted in 3 different orientations, and the average valley-to-peak ratio (VPR) was obtained (19). The resolvability was defined as the percentage of the number of line profiles with VPR under 0.735 over the total number of line profiles for each sector (20).

For imaging performance comparison, the same resolution phantom (^18^F activity of 0.9 MBq) was also scanned by a commercial preclinical PET scanner (Inveon D-PET; Siemens Healthineers) (21) for 30 min. We used an energy window of 350–650 keV, which was an optimized value for rodent imaging using the Inveon PET. We used 2D FBP and 3D OSEM algorithms for analytic and iterative image reconstructions of the Inveon PET data (61.3 million coincidence events), respectively. We used 16 subsets, 20 iterations, and a target resolution of 0.8 mm for the 3D OSEM. For both FBP and OSEM algorithms, we used a zoom factor of 2, which resulted in the voxel size (radial × tangential × axial) of 0.194 × 0.194 × 0.796 mm^3^. The axial slice thickness (0.796 mm) was fixed by the manufacturer.

### Dynamic PET Imaging

For dynamic PET imaging of a mouse brain, we injected ^18^F-FITM tracer (5.0 MBq) into a 7-wk-old, 29 g male Slc:ddy mouse via the tail vein under anesthetized conditions with 1.5%–2.0% isoflurane. The mouse brain was scanned by the HR-PET every 5 min from the injection until 85 min postinjection. The scan time was 5 min for each time point. For image reconstruction, we used the OSEM algorithm with 8 subsets, 1 iteration, and a 3D gaussian image domain blurring kernel size of 1 mm. The voxel size (radial × tangential × axial) was 0.2 × 0.2 × 0.2 mm^3^.

For analysis of the dynamic PET images, we used PMOD software (version 4.303) which provides a template of the mouse brain atlas (Ma-Benveniste-Mirrione) with various predefined volumes of interest (Supplemental Fig. 2; Supplemental Table 2) (22). The time–activity curves of various mouse brain regions were obtained by calculating the average voxel intensity for each region. The physical decay of the ^18^F isotope was corrected for each time–activity curve.

### Comparison of In Vivo PET Imaging and Autoradiography

We injected ^18^F-FITM tracer (8.3 MBq) into a 7-wk-old, 33.9 g male Slc:ddy mouse via the tail vein during an awake state. The mouse brain was scanned by the HR-PET for 30 min with an energy window of 440–560 keV at 40 min postinjection under anesthesia with 1.5%–2.0% isoflurane. The HR-PET data (2.3 million coincidence events) were reconstructed using the OSEM algorithm with 8 subsets, 10 iterations, and a 3D gaussian image domain blurring kernel size of 0.75 mm. The voxel size (radial × tangential × axial) was 0.2 × 0.2 × 0.2 mm^3^.

Subsequently, the mouse was also scanned by the Inveon PET (21) for 10 min with an energy window of 350–650 keV at 75 min postinjection. The Inveon PET data (48.6 million coincidence events) were reconstructed using the 3D OSEM followed by the maximum posterior algorithm with 16 subsets, 8 iterations, and a target resolution of 0.8 mm. The voxel size (radial × tangential × axial) was 0.194 × 0.194 × 0.796 mm^3^.

At 94 min postinjection, the mouse was sacrificed and the brain was removed (Supplemental Fig. 3A). The mouse brain was frozen using powdered dry ice for 5 min and cut into 0.02-mm-thick coronal slices with a cryostat (CryoStar NX70; Thermo Fisher Scientific). The brain slices at the thalamus (−1.46 mm from Bregma) and cerebellum (−6.24 mm from Bregma) levels were covered with an imaging plate (BAS-2025; Fuji Film) and exposed for 45 min at 140 min postinjection (Supplemental Figs. 3B and 4). The imaging plate was read by a reading system (BAS-5000; Fuji Film) with a pixel size of 0.02 × 0.02 mm^2^ (23).

For various mouse brain regions, regions of interest were drawn, and the mean intensities of each mouse brain region obtained with the HR-PET and Inveon PET were compared with the autoradiography.

All animal experiments were conducted in line with the animal experiment guidelines of the National Institutes for Quantum Science and Technology after approval by the local ethical committee of the institute.

## RESULTS

### Resolution Phantom Images

**FIGURE 2.**
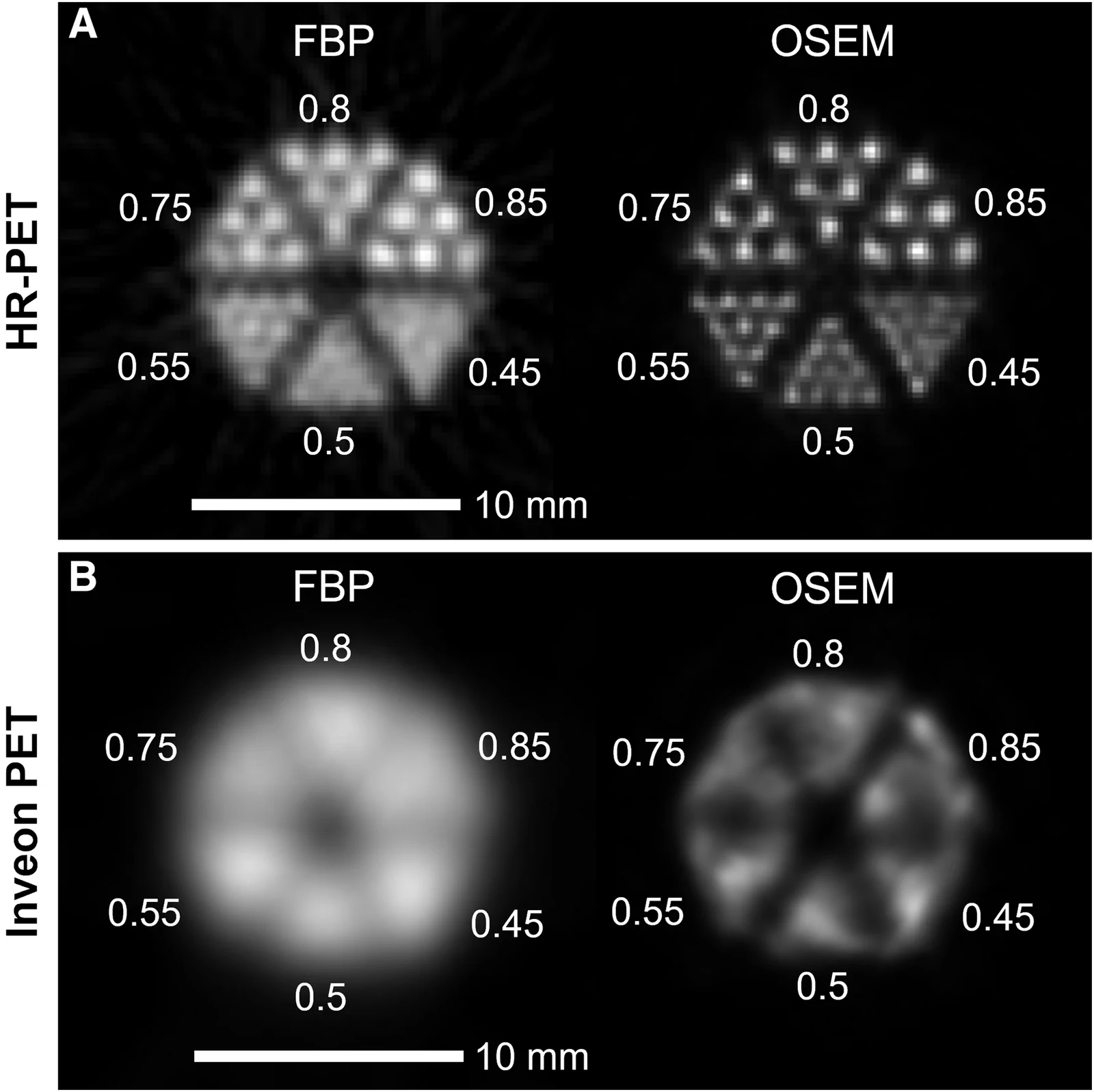
(A) Reconstructed PET images of resolution phantom with HR-PET using FBP (left) and OSEM (right) algorithms. (B) Reconstructed PET images of same resolution phantom with Inveon PET using FBP (left) and OSEM (right) algorithms.

The HR-PET resolved all the rods of the resolution phantom including the 0.45-mm rod diameter structure when using OSEM (Fig. 2A). The average VPRs of each rod sector with OSEM and FBP are summarized (Supplemental Table 3). In contrast, the Inveon PET could not resolve the same rod structures because of the limited spatial resolution (Fig. 2B). Moreover, the ^18^F activity distributions of the rod structures were distorted with the OSEM algorithm compared with those of FBP algorithm when using the Inveon PET.

### Dynamic PET Analysis Results

**FIGURE 3.**
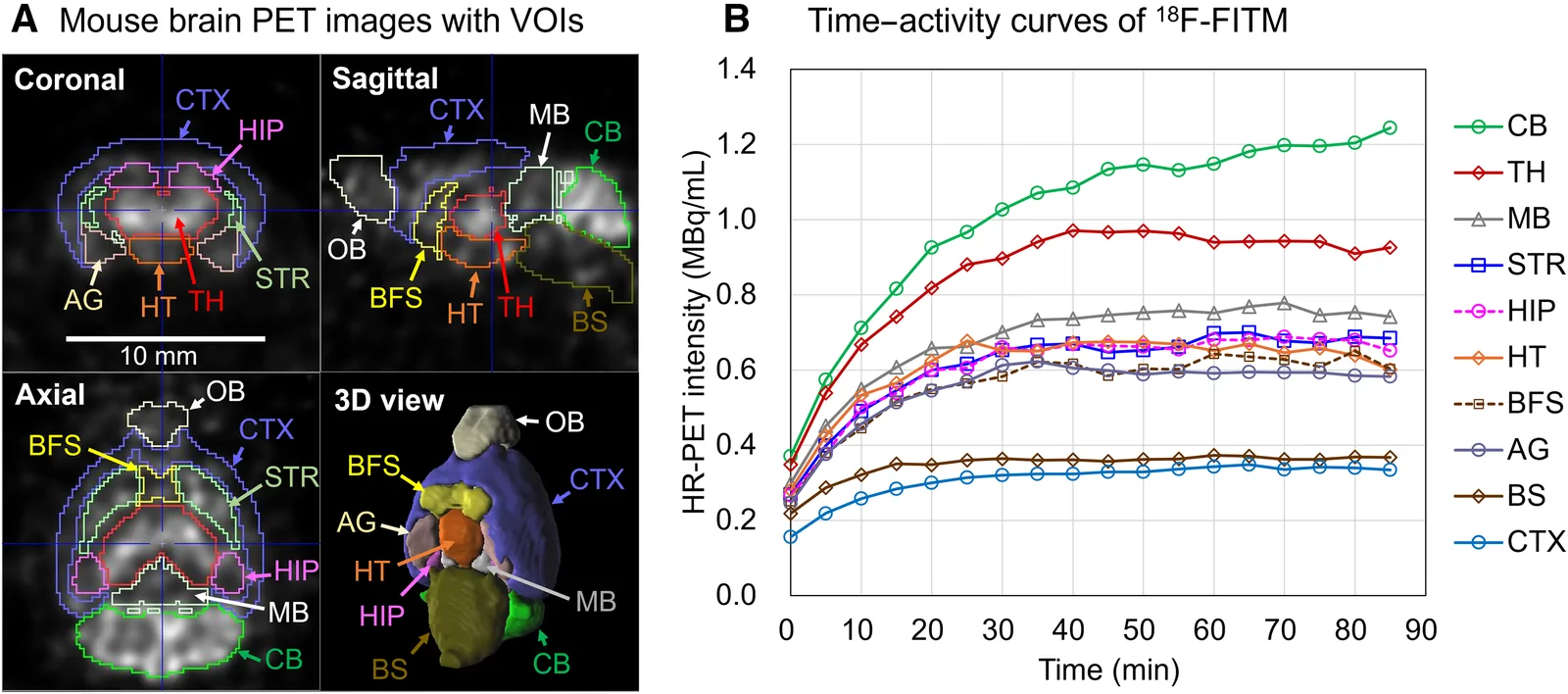
(A) Volumes of interest (VOIs) of various mouse brain regions overlaid with PET images obtained by HR-PET. 3D rendered image of VOIs is also shown. (B) Time–activity curves of ^18^F-FITM uptake at various mouse brain regions. AG = amygdala; BFS = basal forebrain septum; BS = brainstem; CB = cerebellum; CTX = cortex; HIP = hippocampus; HT = hypothalamus; MB = midbrain; STR = striatum; TH = thalamus.

We obtained the time–activity curves of various mouse brain regions with HR-PET (Fig. 3). The cerebellum showed the highest uptake of ^18^F-FITM compared with other regions. The cerebellum uptake rapidly increased to 50 min postinjection and then steadily increased even after 80 min postinjection. The uptake of ^18^F-FITM at the striatum, thalamus, hypothalamus, and hippocampus increased rapidly until 40 min postinjection and reached a plateau at around 60 min postinjection. The uptake of ^18^F-FITM at the cortex and brainstem was almost twice as low as that of the striatum and midbrain regions.

### Mouse Brain Images Obtained with PET and Autoradiography

**FIGURE 4.**
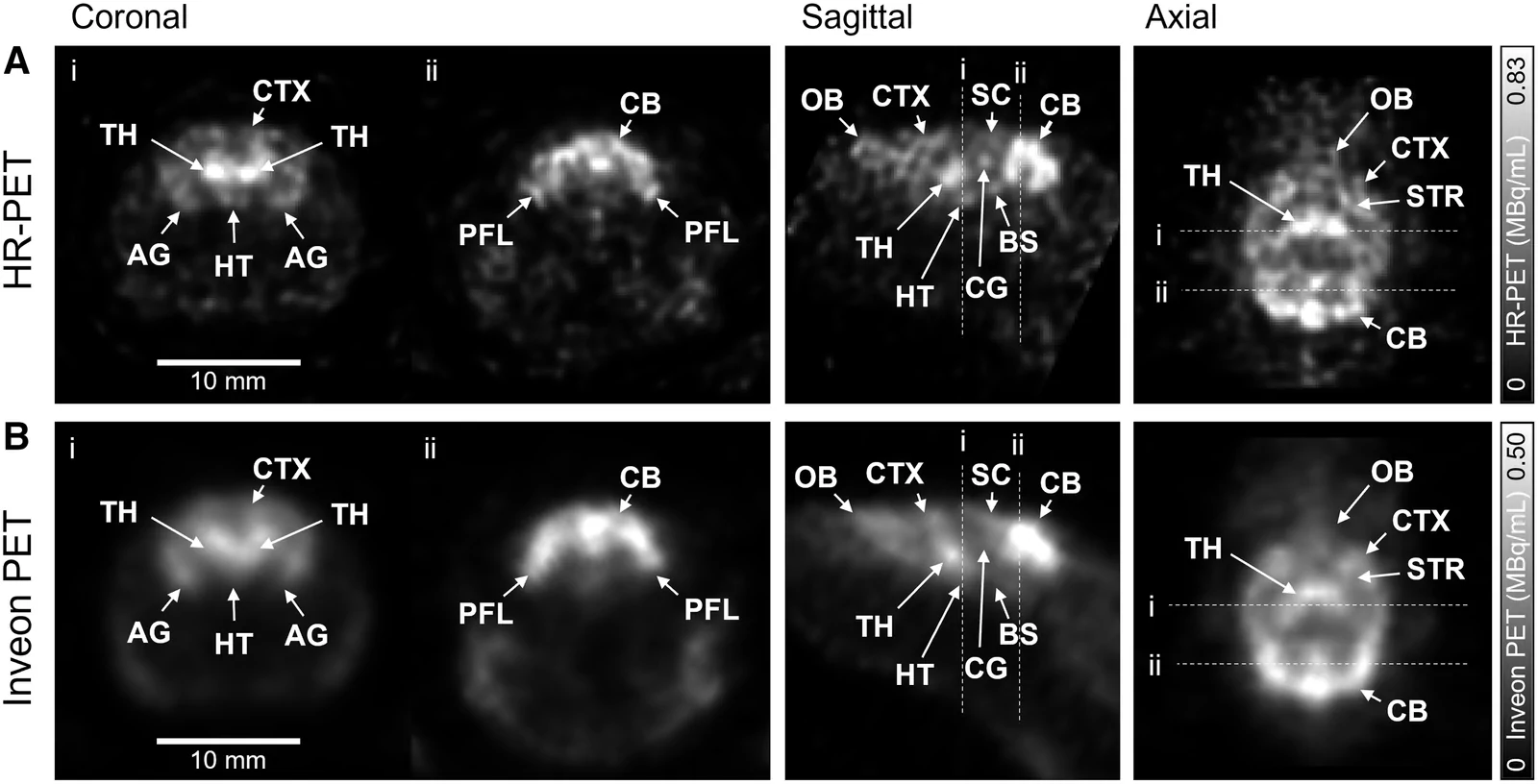
(A) Mouse brain images obtained by HR-PET at 40 min postinjection using OSEM (10 iterations). (B) Mouse brain images obtained by Inveon PET at 75 min postinjection using OSEM (10 iterations). Coronal images were obtained at two different brain levels of thalamus (Bregma, −2.06 mm) and cerebellum (Bregma, −6.24 mm) as indicated by dotted lines (i and ii) in sagittal and axial images. AG = amygdala; BS = brainstem; CB = cerebellum; CG = central gray; CTX = cortex; HT = hypothalamus; OB = olfactory bulb; SC = superior colliculus; STR = striatum; PFL = paraflocculus; TH = thalamus.

HR-PET visualized various mouse brain regions such as thalamus, hypothalamus, amygdala, paraflocculus, central gray, and superior colliculus with the OSEM algorithm (Fig. 4A). Even with the FBP algorithm, HR-PET identified the thalamus and hypothalamus (Supplemental Fig. 7A). In contrast, the same mouse brain structures were barely distinguished when using the Inveon PET (Fig. 4B; Supplemental Fig. 7B).

**FIGURE 5.**
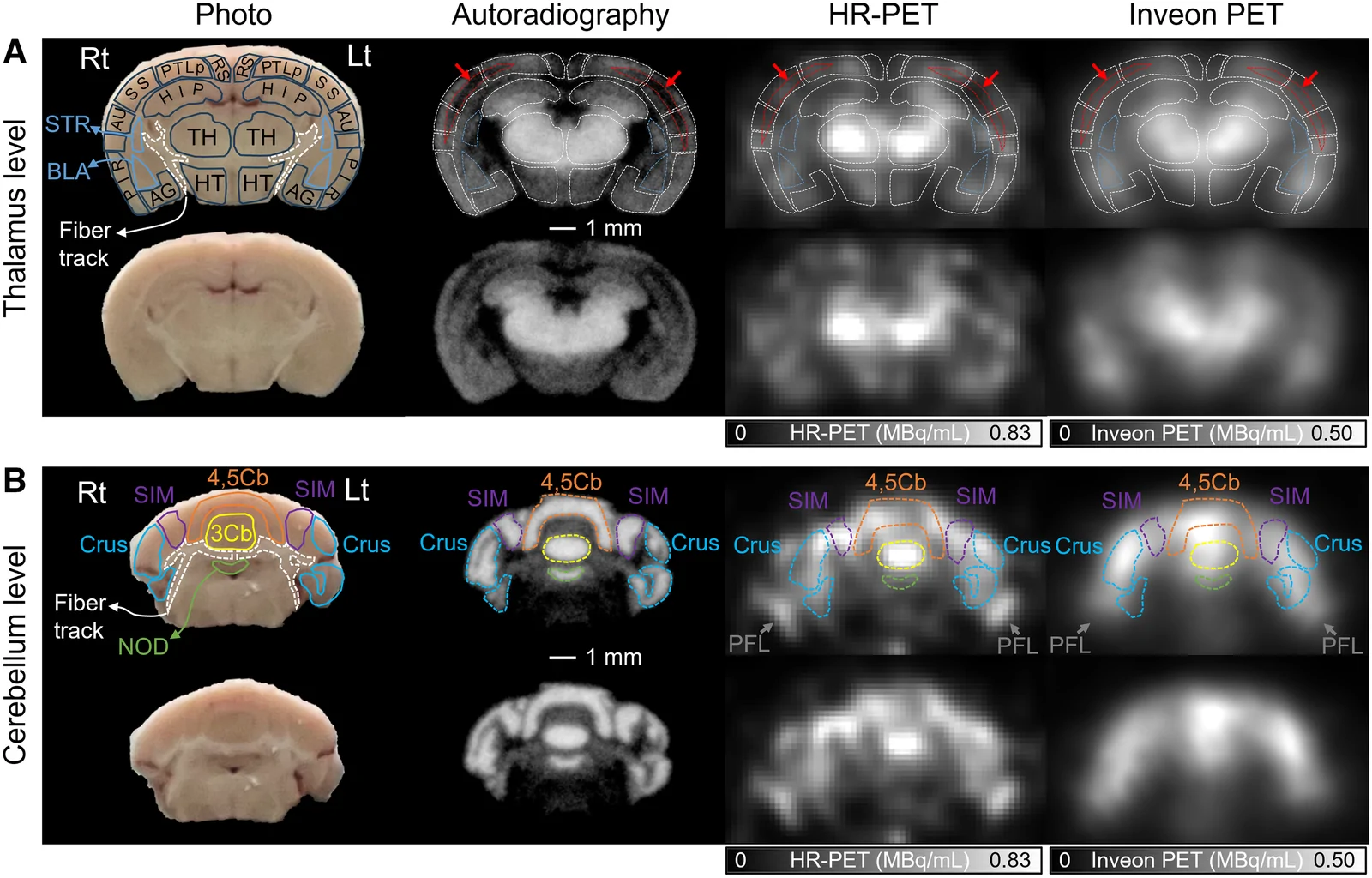
Mouse brain images obtained by photography, autoradiography, HR-PET, and Inveon PET at thalamus level (A) and cerebellum level (B). AG = amygdala; AU = auditory area; BLA = basolateral amygdala; 3Cb = third central lobule; 4,5Cb = fourth and fifth lobules; HIP = hippocampus; HT = hypothalamus; NOD = nodulus; RS = retrosplenial area; SIM = simple lobule; SS = somatosensory area; STR = striatum; PFL = paraflocculus; PIR = piriform; PTLp = posterioparietal association areas; TH = thalamus.

The photos, autoradiography, and HR-PET images of the mouse brain are shown with and without the outlines of various brain areas (Fig. 5). At the thalamus level, autoradiography revealed low uptake of ^18^F-FITM in the cortical layers (indicated by the red arrows) along the auditory, somatosensory, posterior parietal association, and retrosplenial areas (Fig. 5A). The relatively low uptake at the auditory, somatosensory and retrosplenial areas was also identified with the HR-PET. Additionally, the autoradiography clearly visualized the subcortical structures such as hippocampus, thalamus, hypothalamus, amygdala, basolateral amygdala, striatum, and piriform area, which were also identified with the HR-PET but with relatively low resolution. The Inveon PET was hardly able to identify the amygdala and hypothalamus, which are smaller than 1 mm.

At the cerebellum level, autoradiography visualized the high uptake of ^18^F-FITM in the Purkinje cell layer and molecular layer of the crus, simple lobule, fourth and fifth lobules, third central lobule, and nodulus (Fig. 5B). The HR-PET resolved the fourth and fifth lobules and third central lobule; however, the nodulus was not identified because of the limited resolution at the short distance between the third central lobule and nodulus (0.25 mm). The HR-PET identified the paraflocculus on the right and left sides, which were not identified with autoradiography because of the detachment of the paraflocculus during the removal of the mouse brain. The Inveon PET was barely able to distinguish the paraflocculus, the third central lobule, and the fourth and fifth lobules.

**FIGURE 6.**
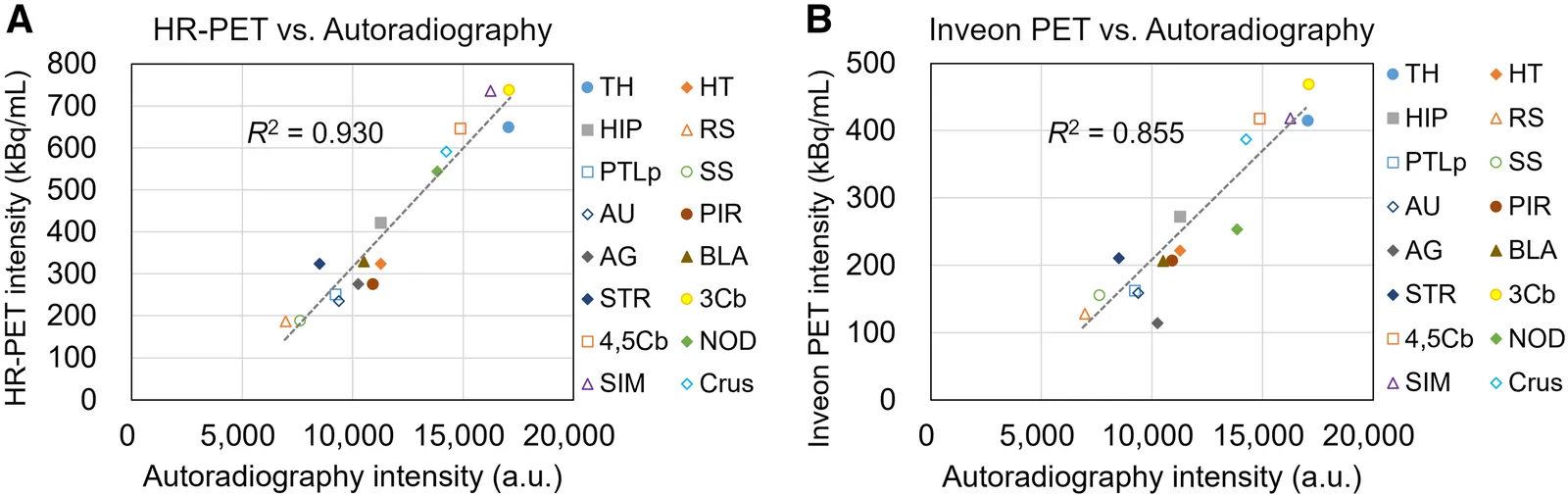
(A) Correlation between HR-PET and autoradiography results. (B) Correlation between Inveon PET and autoradiography results. Linear fitting lines are indicated by dashed lines. AG = amygdala; AU = auditory area; BLA = basolateral amygdala; 3Cb = third central lobule; 4,5Cb = fourth and fifth lobules; HIP = hippocampus; HT = hypothalamus; NOD = nodulus; RS = retrosplenial area; SIM = simple lobule; SS = somatosensory area; STR = striatum; PFL = paraflocculus; PIR = piriform; PTLp = posterioparietal association areas; TH = thalamus.

The HR-PET provided a better correlation with the autoradiography results than did the Inveon PET for various brain regions (Fig. 6). The Inveon PET underestimated the ^18^F-FITM uptake at small brain structures, such as the amygdala and nodulus, because of the partial-volume effect caused by the low spatial resolution (24).

## DISCUSSION

We compared the imaging performance between the HR-PET and Inveon PET using the same resolution phantom and the same scan time (30 min). The HR-PET resolved the 0.45-mm rod diameter structure with an average VPR of 0.679 and resolvability of 91.7% using OSEM with 40 iterations (Fig. 2; Supplemental Table 3). The same rod structure was not resolved with the Inveon PET because of the limited spatial resolution (1.3–1.5 mm) (21). Moreover, unlike the HR-PET, the reconstructed PET images with the Inveon PET were substantially distorted when using high iterations of over 10 (Supplemental Fig. 5). This can be explained by the artificially enhanced spatial resolution at high iterations for a point source in air (25). Therefore, we used 10 iterations for OSEM image reconstruction of in vivo mouse brain data with the Inveon PET (Supplemental Fig. 6). It is worth noting that even at relatively low iterations (i.e., from 3 to 10), the ^18^F activity distributions in the submillimeter rod structures were slightly distorted because of the limited spatial resolution of the Inveon PET.

Previously, dynamic changes of ^18^F-FITM uptake at mGluR1 were investigated mostly with rat models (1,2) rather than mouse models because of the limited spatial resolution of commercial PET scanners. With the HR-PET, dynamic changes of mGluR1 at various mouse brain regions were obtained using ^18^F-FITM (Fig. 3). The cerebellum uptake increased even after 60 min postinjection, which agreed with the previous dynamic PET imaging results using rat models (2). As a next step, we plan to investigate the dynamic changes of ^18^F-FITM uptake at various mouse brain regions using a mouse model of Alzheimer disease.

The HR-PET visualized small mouse brain regions such as the amygdala, hypothalamus, and central gray in coronal and sagittal planes when using the OSEM algorithm (Fig. 4A). Even with the FBP algorithm, the amygdala and hypothalamus were identified (Supplemental Fig. 7A). In contrast, the same mouse brain structures were barely identified with the Inveon PET using OSEM (Fig. 4B) and FBP (Supplemental Fig. 7B).

The HR-PET visualized the high concentrations of mGluR1 at the thalamus with distributions similar to those of the autoradiography (Fig. 5A). The concentration patterns between the HR-PET and Inveon PET may be slightly different because of the different postinjection for each scan (HR-PET, 40 min; Inveon PET, 75 min), especially for the cerebellum and thalamus regions (Fig. 3). The HR-PET identified small cerebellar structures, which were barely resolved with the Inveon PET (Fig. 5B). However, we observed slightly different distributions of ^18^F-FITM uptake between the HR-PET and autoradiography for the crus, simple lobule, and fourth and fifth lobules at the cerebellum level. This is mainly because of a 10-times larger slice thickness used for the HR-PET (0.2 mm) than used with the autoradiography (0.02 mm), as cerebellum structures change even with a 0.1 mm or less interval (Supplemental Fig. 8). Therefore, we plan to generate 3D images of a mouse brain from autoradiography images (26) for quantitative comparison with the HR-PET images over the entire mouse brain. For this task, a silicon detector–based 2D (27) or 3D (28) digital autoradiography technique would be a good solution to save labor and imaging time.

The HR-PET provided a higher correlation with the autoradiography than did the Inveon PET, due to the sub–0.5-mm resolution, which was obtained using optimized DOI detector design parameters, such as crystal pitch (0.8 mm) (15), crystal layer thickness (3 + 3 + 5 mm) (16), and reflector material (diffusive reflector) (17).

One limitation of this study is the lack of statistical comparison between the HR-PET, Inveon PET, and autoradiography, because only a single mouse was used. Therefore, we plan to use multiple mice for statistical comparison of the mGluR1 distributions among the 3 different imaging systems.

The second limitation is that the spatial distribution of mGluR1 was not compared quantitatively among the HR-PET, Inveon PET, and autoradiography. Therefore, we also plan to compare the spatial distribution of mGluR1 concentration inside a mouse brain between the HR-PET and autoradiography with the same axial slice thickness. The structural similarity index (29) would be a good measure to compare the spatial distribution between the HR-PET and autoradiography.

## CONCLUSION

The HR-PET provided ^18^F-FITM uptake images of the mouse brain similar to those of the autoradiography due to to sub–0.5-mm resolution. The HR-PET will open up new opportunities for research into neurodegenerative diseases and disorders in mouse models using radiotracers.

## DISCLOSURE

This work was supported by KAKENHI grants (23K17239, 25K21601), QST President’s Grant (R6 Shorei Kenkyu), Nakatani Foundation, Japan Agency for Medical Research and Development under grant number 24zf0127012, and the 2023 IEEE NPSS Edward J. Hoffman Early Career Development Grant. No other potential conflict of interest relevant to this article was reported.

## Notes

KEY POINTS **QUESTION:** Can the HR-PET visualize the central nervous system of a mouse brain with resolution close to that of autoradiography for neuroscience research? **PERTINENT FINDINGS:** The HR-PET visualized the ^18^F-FITM uptake of a mouse brain with high correlations to uptake obtained by autoradiography. The HR-PET identified the amygdala and cerebellum structures, which were barely delineated when using a commercial preclinical PET scanner. **IMPLICATIONS FOR PATIENT CARE:** The HR-PET will open up new opportunities for translational research and promote new drug discoveries for treatment of neurodegenerative diseases.
